# Supplementary figures and images for: Rhizosphere microbial community structure in high-producing, low-input switchgrass families
Source: PLoS One. 2024 Oct 3;19(10):e0308753. doi: 10.1371/journal.pone.0308753 (PMC11449334; doi:10.1371/journal.pone.0308753)

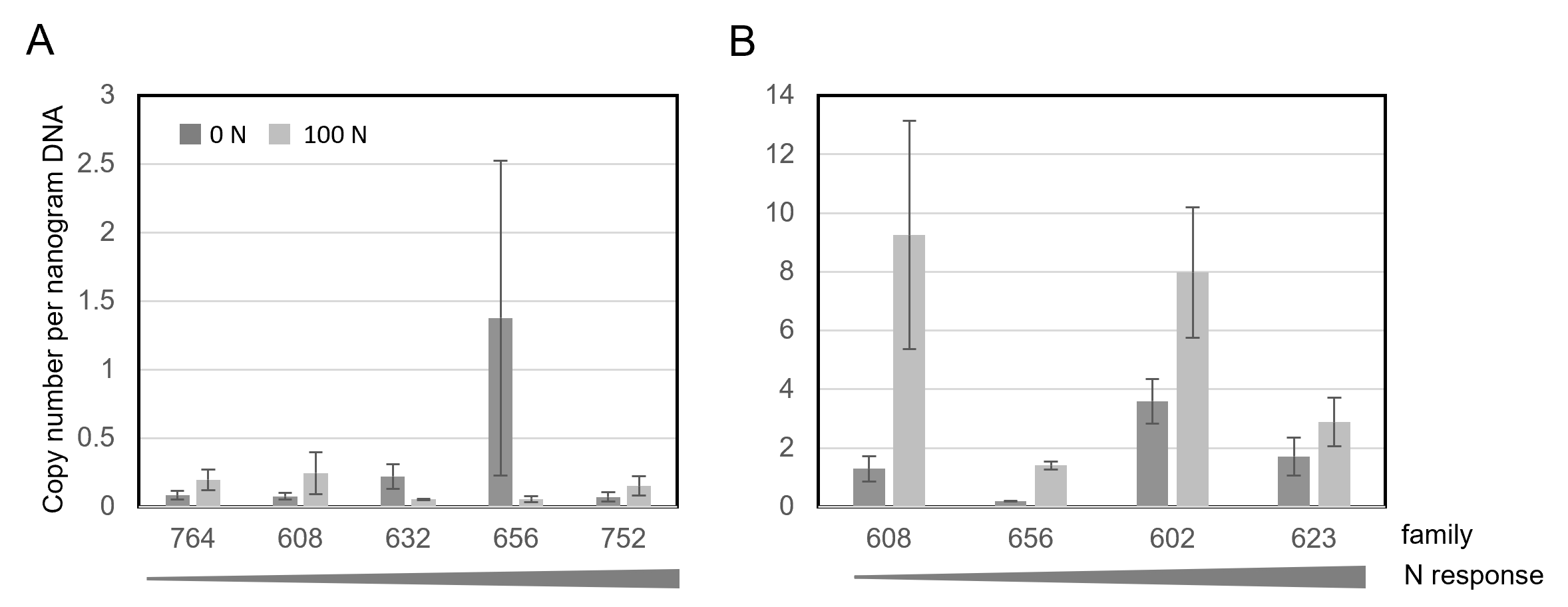

Supplement: S1 Fig — (A) Copy number of AMT1 per nanogram of rhizosphere DNA of five different switchgrass families grown under 0 N and 100 N in Prairie du Sac, Wisconsin (p > 0.1, Student’s t-test). (B) Copy number of AMT1 per nanogram of rhizosphere DNA of four different switchgrass families grown under 0 N and 100 N in Hancock, Wisconsin (p = 0.087, Student’s t-test). Error bars are standard error of the calculated copy number of AMT1. The families on the x-axis are arranged from least responsive to nitrogen to most responsive calculated from percent yield difference. Dark gray bars are families grown with 0 nitrogen; light gray bars are the same families grown with 100 kg/ha nitrogen. (TIF) [file pone.0308753.s001.tif]
