# Supplementary material for: Rhizosphere microbial community structure in high-producing, low-input switchgrass families
Source: PLoS One. 2024 Oct 3;19(10):e0308753. doi: 10.1371/journal.pone.0308753 (PMC11449334; doi:10.1371/journal.pone.0308753)
Supplement: S1 Table — Plants were chosen for sampling and analysis from Casler, 2023. (PDF) [file pone.0308753.s002.pdf]

	Mean	Variance	t-value	p-value
PDS				
Non-responsive	-1.35	1.02	4.86	<0.0001
Responsive	1.03	0.50		
HAN				
Non-responsive	-0.74	0.25	3.69	<0.001
Responsive	0.87	0.35		
