# Supplementary material for: Rhizosphere microbial community structure in high-producing, low-input switchgrass families
Source: PLoS One. 2024 Oct 3;19(10):e0308753. doi: 10.1371/journal.pone.0308753 (PMC11449334; doi:10.1371/journal.pone.0308753)
Supplement: S2 Table — (PDF) [file pone.0308753.s003.pdf]

Primer name	Primer Sequence (5’ to 3’)	Use
ITS_fusion_F	ACACTCTTTCCCTACACGACGCTCTTCCGATCTCTTGGTCATTTAGAGGAAGTAA	ITS fusion primer for Illumina metagenomic sequencing
ITS_fusion_R	GTGACTGGAGTTCAGACGTGTGCTCTTCCGATCTTCCTCCGCTTATTGATATGC	ITS fusion primer for Illumina metagenomic sequencing
Illumina_seq_F	AATGATACGGCGACCACCGAGATCTACAC[55555555]ACACTCTTTCCCTACACGACGCTCTTCCGATCT	Primers to add Illumina dual indexes and sequencing adaptors for metagenomic sequencing
Illumina_seq_R	CAAGCAGAAGACGGCATACGAGAT[77777777]GTGACTGGAGTTCAGACGTGTGCTCTTCCGATCT	Primers to add Illumina dual indexes and sequencing adaptors for metagenomic sequencing
IGK3	GCIWTHTAYGGIAARGGIGGIATHGGIAA	*nifH* qPCR
DVV	ATIGCRAAICCICCRCAIACIACRTC	*nifH* qPCR
AMT1F	AGCCATASCAGCACGAGAWGTAG	*AMT1* qPCR
AMT1R	TGGTGGTACKCCYGTMCACA	*AMT1* qPCR
