# Supplementary material for: Rhizosphere microbial community structure in high-producing, low-input switchgrass families
Source: PLoS One. 2024 Oct 3;19(10):e0308753. doi: 10.1371/journal.pone.0308753 (PMC11449334; doi:10.1371/journal.pone.0308753)
Supplement: S1 File — (PDF) [file pone.0308753.s004.pdf]

S1 File. *nifH* copy number per sample.

plot-family	site	nitrogen_treatment_kg_ha-1	nifH_copies_ng-1_DNA
B10-604	HCK	0	108.6273507
B10-604	HCK	100	47.46152416
B10-623	HCK	0	109.4417719
B10-623	HCK	100	132.9885195
B11-624	HCK	0	31.49106959
B11-624	HCK	100	19.1137143
B11-685	HCK	0	
B11-685	HCK	100	17.70844776
B12-611	HCK	0	60.64633716
B12-611	HCK	100	21.72275483
B12-642	HCK	0	34.25499484
B12-642	HCK	100	158.4775392
B13-661	PdS	0	23.56256627
B13-661	PdS	100	42.29285803
B13-667	PdS	0	53.0234809
B13-667	PdS	100	27.25125306
B14-734	PdS	0	343.5177225
B14-734	PdS	100	120.2630037
B14-752	PdS	0	38.70246734
B14-752	PdS	100	108.3292358
B15-694	PdS	0	233.4191887
B15-694	PdS	100	86.78129326
B15-759	PdS	0	115.6137357
B15-759	PdS	100	19.17258902
B1-634	HCK	0	40.58614318
B1-634	HCK	100	104.4739939
B16-630	PdS	0	85.72852358
B16-630	PdS	100	594.9141617
B16-707	PdS	0	108.1259915
B16-707	PdS	100	74.50167693
B1-766	HCK	0	45.87513516
B1-766	HCK	100	32.36182207
B17-732	PdS	0	73.73022648
B17-732	PdS	100	114.134312
B17-764	PdS	0	45.64895772
B17-764	PdS	100	40.96501016
B18-645	PdS	0	62.77447097
B18-645	PdS	100	16.18514566
B18-652	PdS	0	6.215234062
B18-652	PdS	100	
B19-610	PdS	0	130.1283721
B19-610	PdS	100	51.41338316
B19-688	PdS	0	44.48816137
B19-688	PdS	100	16.89797206
B20-632	PdS	0	100.3388146

B20-632	PdS	100	145.795753
B20-723	PdS	0	140.7981667
B20-723	PdS	100	115.0814255
B21-669	PdS	0	5.058420927
B21-669	PdS	100	55.9429859
B21-743	PdS	0	81.49473274
B21-743	PdS	100	218.1589847
B22-656	PdS	0	38.83874157
B22-656	PdS	100	127.2523971
B22-684	PdS	0	52.75363096
B22-684	PdS	100	117.9905912
B23-741	PdS	0	60.94777483
B23-741	PdS	100	45.93693714
B23-773	PdS	0	14.52551204
B23-773	PdS	100	39.72147358
B24-608	PdS	0	
B24-608	PdS	100	16.10845355
B24-614	PdS	0	364.2983026
B24-614	PdS	100	21.4536897
B2-608	HCK	0	18.48937133
B2-608	HCK	100	50.63480304
B2-663	HCK	0	41.12214992
B2-663	HCK	100	48.79559861
B3-633	HCK	0	265.1967249
B3-633	HCK	100	50.33158205
B3-656	HCK	0	56.53652029
B3-656	HCK	100	52.03962561
B4-656	HCK	0	66.51141572
B4-656	HCK	100	62.39255915
B4-729	HCK	0	46.16201098
B4-729	HCK	100	54.0317506
B5-630	HCK	0	74.3867922
B5-630	HCK	100	38.04430771
B5-654	HCK	0	5.999160892
B5-654	HCK	100	166.3030468
B6-643	HCK	0	36.71173394
B6-643	HCK	100	398.3782114
B6-670	HCK	0	41.24772535
B6-670	HCK	100	16.27385511
B7-633	HCK	0	51.44124603
B7-633	HCK	100	87.31838291
B7-646	HCK	0	57.6982804
B7-646	HCK	100	159.6974655
B8-637	HCK	0	32.82382952
B8-637	HCK	100	20.5362899
B8-657	HCK	0	1094.588278
B8-657	HCK	100	90.54740372

B9-602	HCK	0	182.5199062
B9-602	HCK	100	14.71599599
B9-615	HCK	0	39.8279849
B9-615	HCK	100	62.1029373
