# Supplementary material for: Rhizosphere microbial community structure in high-producing, low-input switchgrass families
Source: PLoS One. 2024 Oct 3;19(10):e0308753. doi: 10.1371/journal.pone.0308753 (PMC11449334; doi:10.1371/journal.pone.0308753)
Supplement: Attachment — Submitted filename: Response to reviewers.pdf [file pone.0308753.s006.pdf]

Mayank Gururani and reviewers:

Thank you for your time and effort to improve this manuscript titled, “Rhizosphere microbial community structure in high-producing, low-input switchgrass families.” We sincerely appreciate the opportunity to edit this manuscript and we hope that we have addressed the comments to the satisfaction of the editors and reviewers. Here, we have included a point-by-point response to all of the comments that we have received. Changes are outlined here and in our marked-up copy of the manuscript.

<u>Editor comments:</u>

When submitting your revision, we need you to address these additional requirements.

1. Please ensure that your manuscript meets PLOS ONE's style requirements, including those for file naming.

> Author response: Thank you for highlighting this. We have checked that our manuscript meets PLOS ONE’s style requirement.

2. Please note that funding information should not appear in any section or other areas of your manuscript. We will only publish funding information present in the Funding Statement section of the online submission form. Please remove any funding-related text from the manuscript.

> Author response: Thank you for pointing this out. We have removed the funding statement from the manuscript.

3. Thank you for stating the following financial disclosure:
"This research was based upon work supported in part by the Great Lakes Bioenergy Research Center, U.S. Department of Energy, Office of Science, Office of Biological and Environmental Research under Award Numbers DE-SC0018409. The research was conducted while the author was an employee of USDA-ARS and partly funded by congressionally allocated funds to the U.S. Dairy Forage Research Center."

Please state what role the funders took in the study. If the funders had no role, please state: "The funders had no role in study design, data collection and analysis, decision to publish, or preparation of the manuscript."
If this statement is not correct you must amend it as needed.
Please include this amended Role of Funder statement in your cover letter; we will change the online submission form on your behalf.

> Author response: Thank you for pointing this out. We wish to add the following to the Funding Statement: "The funders had no role in study design, data collection and analysis, decision to publish, or preparation of the manuscript."

4. When completing the data availability statement of the submission form, you indicated that you will make your data available on acceptance. We strongly recommend all authors decide on a data sharing plan before acceptance, as the process can be lengthy and hold up publication

timelines. Please note that, though access restrictions are acceptable now, your entire data will need to be made freely accessible if your manuscript is accepted for publication. This policy applies to all data except where public deposition would breach compliance with the protocol approved by your research ethics board. If you are unable to adhere to our open data policy, please kindly revise your statement to explain your reasoning and we will seek the editor's input on an exemption. Please be assured that, once you have provided your new statement, the assessment of your exemption will not hold up the peer review process.

Author response: Thank you very much for this suggestion. We have submitted our data to Zenodo and it is currently being reviewed. Here is the URL to access the archive after it has been processed: 10.5281/zenodo.11169989. We have also added it to the Data availability statement (page 16).

5. Please review your reference list to ensure that it is complete and correct. If you have cited papers that have been retracted, please include the rationale for doing so in the manuscript text or remove these references and replace them with relevant current references. Any changes to the reference list should be mentioned in the rebuttal letter that accompanies your revised manuscript. If you need to cite a retracted article, indicate the article's retracted status in the References list and also include a citation and full reference for the retraction notice.

Author response: Thank you for highlighting this. To the best of our knowledge, we have not cited any retracted papers in our manuscript. We have, however, added the following references in order to address some of the reviewer comments (these are also addressed directly in response to the specific comments):

Johnson, Nancy Collins, Gail W. T. Wilson, Jacqueline A. Wilson, R. Michael Miller, and Matthew A. Bowker. "Mycorrhizal Phenotypes and the Law of the Minimum." New Phytologist 205, no. 4 (2015): 1473–84. https://doi.org/10.1111/nph.13172.

Kuzyakov, Y., Friedel, J.K., Stahr, K., 2000. Review of mechanisms and quantification of priming effects. Soil Biol. Biochem. 32, 1485–1498. https://doi.org/10.1016/S0038-0717(00)00084-5

Riley, R.C., Cavagnaro, T.R., Brien, C., Smith, F.A., Smith, S.E., Berger, B., Garnett, T., Stonor, R., Schilling, R.K., Chen, Z.-H., Powell, J.R., 2019. Resource allocation to growth or luxury consumption drives mycorrhizal responses. Ecol. Lett. 22, 1757–1766. https://doi.org/10.1111/ele.13353

Stacey, G., Burris R., Evans H. Biological Nitrogen Fixation. New York, NY: Springer, 1992. https://link.springer.com/book/9780412024214.

<u>Reviewer comments:</u>
Review Comments to the Author

Please use the space provided to explain your answers to the questions above. You may also include additional comments for the author, including concerns about dual publication, research

ethics, or publication ethics. (Please upload your review as an attachment if it exceeds 20,000 characters)

Reviewer #1: Dear authors,

this is an interesting work and can be published in this journal.

please present your Rescripts for diversity analyses (phyloseq,..) in supplementary file (GitHub link) and refer in the materials and methods.

Best regard

> Author response: Thank you very much for your thoughtful feedback on this manuscript. We have added our R scripts to the same repository that we have made our data publicly available (10.5281/zenodo.11169989). We think that this will make accessing our data and scripts streamlined and efficient. We added a reference to the location of our R scripts to the manuscript as well (page 10, line 221).

Reviewer #2: The MS by Stonoha-Arther et. al investigates microbial communities of switchgrass rhizosphere in response to nitrogen availibility in two different locations.The study identifies unique microbial taxa associated with nitrogen non-responsive switchgrass families and highlights the complex interactions between plant genotype, location, and soil microbial communities in switchgrass nitrogen response, the leads from which can be adapted for future breeding programs to reduce nitrogen use in switchgrass cultivation. However, the MS in its present forms needs to be revised suitably before accepted for publication. The major and minor comments attached as a word document

> Author response: Thank you very much for your close reading of the manuscript. We have addressed the comments that came in the form of a Word document.

Additional comments from reviewer 2:

Major Comments:

1. The introduction part needs major reaarngements, e.g. the role of microbes and Arbuscular mycorrhizal fungi in general may be concised and brought before throughly summerizring the role of these two in context of switchgrass.

   > Author response: Thank you very much for this useful suggestion. We have substantially edited and rearranged the introduction to increase readability and relevancy. We took the specific suggestion about the paragraphs dedicated to the role of microbes and AMF and made those more concise (pages 3 and 4).

2. The result section is an overlap and contains a lot of methodology, which needs to be suitably edited. Overall the the results are hard to follow throughout due to lack of proper presentation, it needs to elaborated for better readability.

Author response: Thank you very much for pointing this out. We have made significant edits throughout the results section in order to improve its usefulness and flow. We have taken out excess detail that belongs in the methods section (see pages 12 and 13 in the marked-up manuscript for an example).

3. They identified some unique microbial taxa associated with nitrogen non-responders indicating genotype-specific interactions with rhizosphere microbiota. Further functional studies needs to be carried out to validate the mechanisms underlying these associations, as there is no correlation in nifH abundance between nitrogen-responsive and non-responsive groups raising query, if at all, there is any direct involvement of such microbial groups in mediating switchgrass response to nitrogen. Additional functional studies needs to be performed showing the roles of atleast a few specific taxa.

   Author response: Thank you for this comment. We agree that further functional studies on some of the taxa outlined in this manuscript need to be followed up on. Future work will be focused on the possible functions that these taxa are performing for the switchgrass. For this study, we had the following objectives: to characterize the switchgrass rhizosphere microbiome with and without added nitrogen fertilizer; to identify microbial candidates that may contribute to the biomass accumulation of switchgrass genotypes that do not respond to nitrogen fertilizer; and to identify any microbes that may be preventing the non-responsive switchgrass genotypes from utilizing nitrogen fertilizer (page 4). While it outside the scope of this paper to include functional studies, we added the following to the discussion (page 14) to address the *nifH* qPCR results: “However, presence of DNA does not necessarily indicate a difference in expression of the gene. *nifH* gene expression could be higher in the non-responders and receive greater amounts of fixed nitrogen despite the lack of difference in *nifH* copy number or gene abundance.”

4. In one location, there was an increase in *AMT1* gene abundance in response to nitrogen fertilization which is contradictory. Is this due to underlying complexity of plant-microbe interactions along with existing environmental conditions at that specific site? If the authors wish to keep this observation in manuscript, they need to provide logical explaination and hypothesis in support of this.

   Author response: Thank you very much for pointing this out. We think these results are important to include in the paper and have added our hypothesis about what they mean to the discussion (page 15): “However, other studies have demonstrated that AMF can function as commensalists or parasites when plants are grown under nitrogen limiting conditions and the effect was dependent on plant, and, presumably, fungi genotypes [43,44].” These two cited papers were added during the revision. They have been added to the references:

   Johnson, Nancy Collins, Gail W. T. Wilson, Jacqueline A. Wilson, R. Michael Miller, and Matthew A. Bowker. “Mycorrhizal Phenotypes and the Law of the

Minimum." New Phytologist 205, no. 4 (2015): 1473–84. https://doi.org/10.1111/nph.13172.

Riley, R.C., Cavagnaro, T.R., Brien, C., Smith, F.A., Smith, S.E., Berger, B., Garnett, T., Stonor, R., Schilling, R.K., Chen, Z.-H., Powell, J.R., 2019. Resource allocation to growth or luxury consumption drives mycorrhizal responses. Ecol. Lett. 22, 1757–1766. https://doi.org/10.1111/ele.13353

Line 59: Expand AMF at its first use in the text

Author response: Done! Thank you. New line is 52.

Line 79: Cite updated references

Author response: Done! Thank you. New reference is:

Stacey, G., Burris R., Evans H. Biological Nitrogen Fixation. New York, NY: Springer, 1992. https://link.springer.com/book/9780412024214.

Line 89: AMT1 gene name should be italic

Author response: Thank you for noticing this. This was actually taken out during our editing of the introduction.

Line 105: Cite updated references

Author response: Done! Thank you. The reference is:

Kuzyakov, Y., Friedel, J.K., Stahr, K., 2000. Review of mechanisms and quantification of priming effects. Soil Biol. Biochem. 32, 1485–1498. https://doi.org/10.1016/S0038-0717(00)00084-5

Line 110-11: Rephrase the sentence for better context

Author response: Thank you for pointing this out. We have made the following changes (new line 89): "One reason that switchgrass is an attractive bioenergy crop is because it produces biomass on marginal soils. In order to further improve switchgrass for this purpose, it has been suggested that switchgrass should be bred to require less nitrogen while concomitantly selected for increased biomass in order to decrease the nitrogen removal that will inevitably increase if it is bred for biomass only [19]."
