# Supplementary material for: Rhizosphere microbial community structure in high-producing, low-input switchgrass families
Source: PLoS One. 2024 Oct 3;19(10):e0308753. doi: 10.1371/journal.pone.0308753 (PMC11449334; doi:10.1371/journal.pone.0308753)
Supplement: Attachment — Submitted filename: comments.docx [file pone.0308753.s005.docx]

The MS by Stonoha-Arther et. al investigates microbial communities of switchgrass rhizosphere in response to nitrogen availibility in two different locations.The study identifies unique microbial taxa associated with nitrogen non-responsive switchgrass families and highlights the complex interactions between plant genotype, location, and soil microbial communities in switchgrass nitrogen response, the leads from which can be adapted for future breeding programs to reduce nitrogen use in switchgrass cultivation. However, the MS in its present forms needs to be revised suitably before accepted for publication.

Major Comments:

1. The introduction part needs major reaarngements, e.g. the role of microbes and Arbuscular mycorrhizal fungi in general may be concised and brought before throughly summerizring the role of these two in context of switchgrass.
2. The result section is an overlap and contains a lot of methodology, which needs to be suitably edited. Overall the the results are hard to follow throughout due to lack of proper presentation, it needs to elaborated for better readability.
3. They identified some unique microbial taxa associated with nitrogen non-responders indicating genotype-specific interactions with rhizosphere microbiota. Further functional studies needs to be carried out to validate the mechanisms underlying these associations, as there is no correlation in nifH abundance between nitrogen-responsive and non-responsive groups raising query, if at all, there is any direct involvement of such microbial groups in mediating switchgrass response to nitrogen. Additional functional studies needs to be performed showing the roles of atleast a few specific taxa.
4. In one location, there was an increase in *AMT1* gene abundance in response to nitrogen fertilization which is contradictory. Is this due to underlying complexity of plant-microbe interactions along with existing environmental conditions at that specific site? If the authors wish to keep this observation in manuscript, they need to provide logical explaination and hypothesis in support of this.

Line 59: Expand AMF at its first use in the text

Line 79: Cite updated references

Line 89: AMT1 gene name should be italic

Line 105: Cite updated references

Line 110-11: Rephrase the sentence for better context
