# Supplementary material for: Rhizosphere microbial community structure in high-producing, low-input switchgrass families
Source: PLoS One. 2024 Oct 3;19(10):e0308753. doi: 10.1371/journal.pone.0308753 (PMC11449334; doi:10.1371/journal.pone.0308753)
Supplement: Attachment — Submitted filename: response to reviewers second round.docx [file pone.0308753.s007.docx]

Mayank Gururani and reviewers:

Thank you for your time and effort to improve this manuscript titled, “Rhizosphere microbial community structure in high-producing, low-input switchgrass families.” We sincerely appreciate the opportunity to edit this manuscript and we hope that we have addressed the comments to the satisfaction of the editors and reviewers. Here, we have included a point-by-point response to all of the comments that we have received for this second round of revisions. Changes are outlined here and in our marked-up copy of the manuscript.

**Reviewer comments:**
Review Comments to the Author

Please use the space provided to explain your answers to the questions above. You may also include additional comments for the author, including concerns about dual publication, research ethics, or publication ethics. (Please upload your review as an attachment if it exceeds 20,000 characters)

Reviewer #3: The manuscript titled "Rhizosphere Microbial Community Structure in High-Producing, Low-Input Switchgrass Families," authored by Christina Stonoha-Arther et al., provides valuable insights for the scientific community working on enhancing biomass production on marginal land for cost-effective bioenergy and forage production. The information presented in this manuscript will be of great interest to researchers in this field. The manuscript has been thoroughly revised based on previous Reviewer's comments, and it is now ready for acceptance.

Author response: Thank you very much for your careful reading of the manuscript and your thoughts on this study. We sincerely appreciate the time that you took to review our paper and we hope that the scientific community finds this work useful.

Reviewer #4: Major Suggestions:

1.Reorganize the Introduction section: Condense the general information about the role of microbes and arbuscular mycorrhizal fungi (AMF), and focus more on summarizing their specific relevance in the context of switchgrass. This will improve the flow and relevance of the introduction.

Author response: Thank you very much for this useful suggestion. We have substantially edited and rearranged the introduction to increase readability and relevancy. We took the specific suggestion about the paragraphs dedicated to the role of microbes and AMF and made those more concise (pages 3 and 4).

2.Revise the Results section: There is an overlap between the Results and Methodology, which makes the results hard to follow. Separate the methodology details and present the results in a more structured and elaborated manner for better readability.

Author response: Thank you very much for pointing this out. We have made significant edits throughout the results section in order to improve its usefulness and flow. We have taken out excess detail that belongs in the methods section (see pages 12 and 13 in the marked-up manuscript for an example).

3.Perform additional functional studies: The paper identified unique microbial taxa associated with nitrogen non-responsive switchgrass families, suggesting genotype-specific interactions with the rhizosphere microbiota. However, the lack of correlation between nifH abundance and nitrogen responsiveness raises questions about the direct involvement of these microbial groups in mediating switchgrass response to nitrogen. Additional functional studies should be performed to validate the mechanisms underlying these associations and the roles of specific taxa.

Author response: Thank you for this comment. We agree that further functional studies on some of the taxa outlined in this manuscript need to be followed up on. Future work will be focused on the possible functions that these taxa are performing for the switchgrass. For this study, we had the following objectives: to characterize the switchgrass rhizosphere microbiome with and without added nitrogen fertilizer; to identify microbial candidates that may contribute to the biomass accumulation of switchgrass genotypes that do not respond to nitrogen fertilizer; and to identify any microbes that may be preventing the non-responsive switchgrass genotypes from utilizing nitrogen fertilizer (page 4). While it outside the scope of this paper to include functional studies, we added the following to the discussion (page 14) to address the *nifH* qPCR results: “However, presence of DNA does not necessarily indicate a difference in expression of the gene. *nifH* gene expression could be higher in the non-responders and receive greater amounts of fixed nitrogen despite the lack of difference in *nifH* copy number or gene abundance.”

4.Explain the contradictory increase in AMT1 gene abundance: The increase in AMT1 gene abundance in response to nitrogen fertilization at one location is contradictory to the expected trend. Provide a logical explanation and hypothesis to support this observation, considering the underlying complexity of plant-microbe interactions and the environmental conditions at that specific site.

Author response: Thank you very much for pointing this out. We think these results are important to include in the paper and have added our hypothesis about what they mean to the discussion (page 15): “However, other studies have demonstrated that AMF can function as commensalists or parasites when plants are grown under nitrogen limiting conditions and the effect was dependent on plant, and, presumably, fungi genotypes [43,44].”

Minor Suggestions:

1.Expand the abbreviation "AMF" at its first use in the text.

Author response: Done! Thank you. New line is 52.

2.Cite the updated references for lines 79 and 105.

Author response: Done! Thank you. New reference for line 79 is:

Stacey, G., Burris R., Evans H. Biological Nitrogen Fixation. New York, NY: Springer, 1992. https://link.springer.com/book/9780412024214.

The reference for line 105 is:

Kuzyakov, Y., Friedel, J.K., Stahr, K., 2000. Review of mechanisms and quantification of priming effects. Soil Biol. Biochem. 32, 1485–1498. https://doi.org/10.1016/S0038-0717(00)00084-5

3.Rephrase the sentence on lines 110-111 for better context.

Author response: Thank you for pointing this out. We have made the following changes (new line 89): “One reason that switchgrass is an attractive bioenergy crop is because it produces biomass on marginal soils. In order to further improve switchgrass for this purpose, it has been suggested that switchgrass should be bred to require less nitrogen while concomitantly selected for increased biomass in order to decrease the nitrogen removal that will inevitably increase if it is bred for biomass only [19].”
